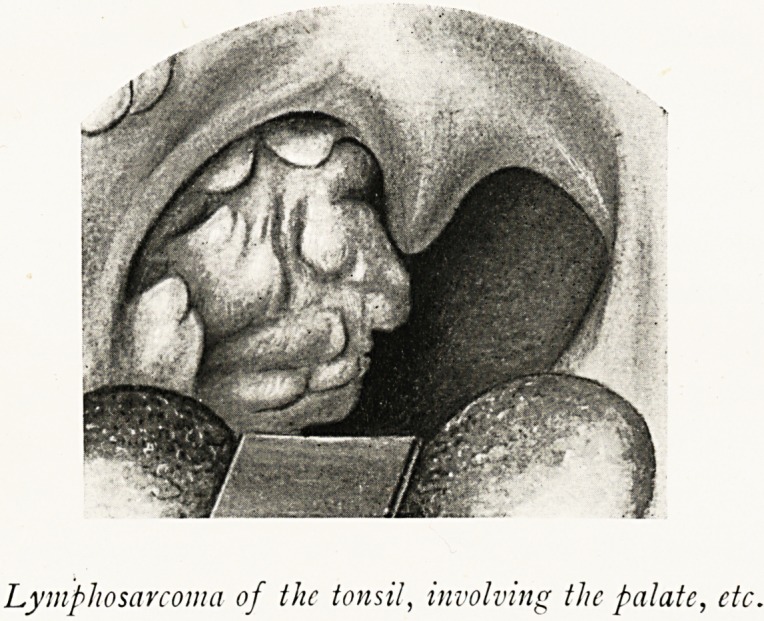# Case of Lymphosarcoma of the Tonsil and Palate

**Published:** 1920-03

**Authors:** P. Watson-Williams

**Affiliations:** Aurist and Laryngologist to the Bristol Royal Infirmary


*Lymphosarcoma of the tonsil, involving the palate, etc.*

### CASE OF LYMPHOSARCOMA OF THE TONSIL AND PALATE, &c.

BY

P. WATSON-WILLIAMS, M.D. Lond.,

*Aurist and Laryngologist to the Bristol Royal Infirmary.*

---

THE value of a radical operation in even somewhat advanced cases of some malignant neoplasms of the tonsils and palate appears exemplified in the case illustrated; and as the desirability of undertaking an extensive operation has to be weighed against the risks involved and the palliative procedures that are open to us, such as injections of selenium or copper alynin, or removal by diathermy, the satisfactory result of a radical operation in this case seems worthy of record.

C. M., male, aged 36, came to the Royal Infirmary on April 24th, 1919, with a large sarcoma originating in the right tonsil and involving the soft palate and the palatal pillars on his right side. The glands in the neck below and behind the right lower jaw and beneath the sternomastoid were considerably enlarged. The patient declared that he had noticed the enlargement for five weeks only, but that it had increased very rapidly.

A portion of the growth was removed and referred to Professor Walker Hall, who reported that "the section shows extensive degeneration and hemorrhages. The connective tissue is very poorly developed. The greater mass of the tissue consists of atypical small cells. These approach the mother cells of lymphoid tissues in type. I regard the tissue as a *lymphosarcoma.*"

*Operation on May 20th.*—The right external carotid artery was ligated and the enlarged glands in the neck carefully and completely removed.

*May 27th.*—The tonsillar growth, together with the palatal pillars, and the right half of the velum removed, including the uvula. The previous ligation of the external carotid artery rendered this operation almost bloodless.

*March 23rd,* 1920.—The patient has increased in weight and has no discomfort. There is no sign of recurrence in the throat or in the glands of the neck.

## Figures and Tables

**Figure f1:**